# Drinking water vulnerability to climate change and alternatives for adaptation in coastal South and South East Asia

**DOI:** 10.1007/s10584-016-1617-1

**Published:** 2016-02-24

**Authors:** M. A. Hoque, P. F. D. Scheelbeek, P. Vineis, A. E. Khan, K. M. Ahmed, A. P. Butler

**Affiliations:** Department of Civil and Environmental Engineering, Imperial College London, South Kensington, London, SW7 2AZ UK; MRC-PHE Centre for Environment and Health, Department of Epidemiology and Biostatistics, Faculty of Medicine, Imperial College London, Norfolk Place, London, W2 1PG UK; Department of Geology, University of Dhaka, Dhaka, Bangladesh

## Abstract

Drinking water in much of Asia, particularly in coastal and rural settings, is provided by a variety of sources, which are widely distributed and frequently managed at an individual or local community level. Coastal and near-inland drinking water sources in South and South East (SSE) Asia are vulnerable to contamination by seawater, most dramatically from tropical cyclone induced storm surges. This paper assesses spatial vulnerabilities to salinisation of drinking water sources due to meteorological variability and climate change along the (ca. 6000 km) coastline of SSE Asia. The risks of increasing climatic stresses are first considered, and then maps of relative vulnerability along the entire coastline are developed, using data from global scale land surface models, along with an overall vulnerability index. The results show that surface and near-surface drinking water in the coastal areas of the mega-deltas in Vietnam and Bangladesh-India are most vulnerable, putting more than 25 million people at risk of drinking ‘saline’ water. Climate change is likely to exacerbate this problem, with adverse consequences for health, such as prevalence of hypertension and cardiovascular diseases. There is a need for identifying locations that are most at risk of salinisation in order for policy makers and local officials to implement strategies for reducing these health impacts. To counter the risks associated with these vulnerabilities, possible adaptation measures are also outlined. We conclude that detailed and fine scale vulnerability assessments may become crucial for planning targeted adaptation programmes along these coasts.

**Electronic supplementary material:**

The online version of this article (doi:10.1007/s10584-016-1617-1) contains supplementary material, which is available to authorised users.

## Introduction

The landscape of SSE Asia is characterised by the Himalayan Mountains, and its associated topography and diverse river systems. The continental rivers that drain the Himalaya and adjacent areas give rise to some of the world’s largest mega-deltas (Bengal, Irrawaddy, Chao Phraya, Mekong and Red River), which are located along the coasts of SSE Asia. This riverine-landscape coupled the mighty Asian monsoons (with distinct wet and dry seasons) provides people with agriculture-based economic opportunities along the coastal regions. The many millions living along these rural coastlines rely on a variety of distributed and individually managed sources (rainwater, surface water, or groundwater) for drinking water. In southwest Bangladesh >70 % of the coastal population is reliant on unprotected sources, such as rain water, rivers, canals and ponds (Islam et al. 2013), and >60 % of rural households consume water from similar sources in the Chao Phraya (UNESCO 2003) and Irrawaddy (Tripartite Core Group 2008) deltas. Along the (South East and South Central) coasts of Vietnam shallow groundwater is the dominant source of drinking water, with ca. 60 % of households provided by hand dug-wells, and a further ca. 25 % of households through drilled wells; the remaining 15 % of the population relying on surface water and rainwater (Snelgrove and Patrick 2009). In contrast, in the Mekong and Red River delta regions >50 % of households rely on surface water and harvested rainwater (Snelgrove and Patrick 2009) because shallow groundwater is unsuitable due to its high iron content and salinity, coupled with an offensive taste and smell (Delta Alliance 2011).

Coastal and near-inland drinking water resources in SSE Asia are vulnerable to contamination from seawater, in particular during episodic inundation events. Such events include: storm surges (mostly due to cyclonic tropical storms); tsunamis (under sea earthquake); inland flooding (due to excessive rainfall), and shallow coastal flooding (due to extreme tide). On a longer time scale, eustatic sea level change (i.e. changes in global mean sea level or local variations due to subsidence and other geological factors) can enhance, both in terms of frequency and/or magnitude, inundation events in coastal areas. Furthermore, climate change is likely to exacerbate these impacts due to sea level rise, increased sea-surface temperature, and more intense rainfall (e.g., Karim and Mimura 2008) The factors that drive these vulnerabilities are both environmental and anthropogenic (Alcamo et al. 2007). Their impacts are particularly pronounced in coastal areas due to a variety of causes, which include: geomorphology, population density, inland modifications of river courses, and withdrawal of upstream water (Syvitski et al. 2009; Hijioka et al. 2014; Saito et al. 2007). Inundation events, notably associated with landfalling cyclones, are among the most frequent, costly, and deadly hazards that can adversely affect coastal communities, particularly in deltas where population density is high and socio-economic conditions are poor.

The aim of this paper is to understand the vulnerabilities of drinking water (sources, and means of collection and storage) to the above hazards, particularly vulnerabilities to salinisation by inundation, and map their relative magnitude along the coastline of SSE Asia. It also seeks to combine the individual components into a single index, which can be used to provide policy makers with a tool for identifying key vulnerable areas. This allows adaptation measures to be prioritised in order to reduce the associated risks to vulnerable resources, thereby protecting, and potentially improving, human health. The paper first describes the hydrometeorological processes that govern water resources in these areas. This leads to a detailed consideration of the individual components that govern water resource vulnerability. A scoring system is adopted, which allows the relative vulnerabilities to be mapped. Combining these scores into a single index provides a simplified method for presenting drinking water vulnerability. Finally, in light of these results, impact of climate change and some adaptation methods are discussed.

## Drinking water sources, means of collection and storage, and inundation risks

In SSE Asia groundwater is the dominant source of drinking water, frequently providing >50 % of the total demand, with the remainder coming from surface water bodies and harvested rainwater (Villholth 2013). Water supplies for domestic and drinking purposes are provided by large-scale water providers in urban areas, and by individual, largely hand pumped tubewells, in rural areas. Away from the coast, the majority of the rural population uses a single source, generally a tubewell, to tap groundwater. The Asian coast has a high potential for groundwater resources due to its unconsolidated to semi-consolidated Quaternary sediments in multi-aquifer settings. However, relatively shallow groundwater (i.e. at depths <150 mbgl) is generally impacted by high natural salinities (van Weert et al. 2009) and naturally occurring arsenic (Ravenscroft et al. 2009) while in some areas deep groundwater (>150 mbgl) is fresh and arsenic free. Due to this high groundwater salinity the coastal population, in contrast, relies on multiple sources (ESM Fig. S4) to meet their drinking water demands. Harvested rainwater, *stored in large jars* (ca. *40 l*), *communal tanks and surface water ponds* at ground level and therefore prone to inundation, plays a vital role in coastal areas. However, once the stored rainwater is used up, or when pond water becomes too saline and/or resource-limited from evaporation losses, people then inevitably rely on relatively saline tubewell and/or river water sources. As tubewells are often completed at, or near to, ground level, these are also vulnerable to inundation. Similarly, hand dug wells, commonly used in the Thailand-Vietnam section of the coastline (Anderson 1978), are often completed at ground level and therefore also vulnerable to inundation.

## Methods and materials

### Identifying variables for vulnerability assessment and mapping

The coastal inundation vulnerability is a function of exposure, sensitivity and resilience to various variables, like elevation, geomorphology, geology, shoreline type, river mouth, orientation, population, rainfall etc. A number of coastal variables are considered in published work, the majority of those range between six to 19 variables (McLaughlin and Cooper 2010). However, increasing the number of variables does not necessarily improve the assessments, since variables can be highly correlated (cf. Balica et al. 2012). For example, geology, shoreline type, river mouth and orientation, all these could be treated under geomorphology due to their similar character. From a vulnerability perspective, the main consequences of coastal inundation arise from its effects on the drinking water sources, and on the means of collection and storage of drinking water. Here a deductive approach is used to identify possible variables based on different relational situations and characteristics of a coastal system exposed to inundation risks.

**Fig. 1:**
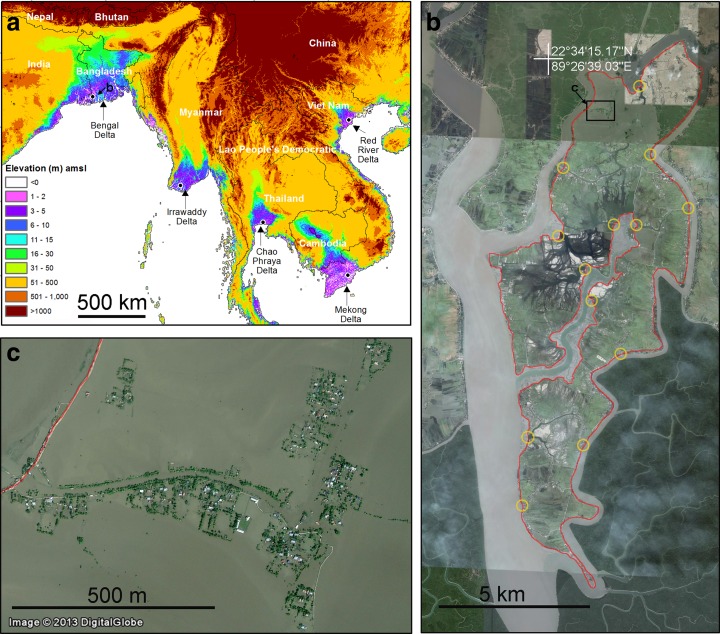
Physical settings of the area. **a** Elevation differences along the SSE Asian coasts, where deltas are characteristically no more than 10 m amsl. **b** A typical coastal landform with polder (red line) and sluice gates (yellow circles) is shown from southern Bangladesh (part of Dacope Upazila). Note that the image is a Google mosaic of images from different dates between 2010 and 2013. A point (white cross) is marked for reference purposes. c) An example of inundation due to failure of a polder is shown. The image date is 11/04/2010 and the inundation is due to cyclone Aila (27 May 2009) induced damage of a polder which was left unfixed until end of 2011. Position of panels ‘b’ and ‘c’ are indicated in panels ‘a’ and ‘b’ respectively

Elevations over SSE Asia vary from less than 1 m above mean sea-level (amsl) near the coast to thousands of metres in the mountainous areas. Over 150 million people live in coastal locations that are less than 5 m amsl. The deltaic regions are typically very low and flat (Fig. 1a) and are characterised by wide (generally >100 m) rivers with their tributaries and distributaries. Coastal regions of these deltas are flat with many tidal rivers and creeks, which experience semi-diurnal tides of a couple of metres amplitude (Singh 2002; Kravtsova et al. 2009). These tidal flats are generally less than 1 to 3 m amsl and can potentially be flooded by tidal water twice a day. Many people in the coastal regions of these deltas live on embanked reclaimed land (Molle and Tuân 2006) locally known as polders (Fig. 1b, and ESM Fig. S3). The tops of these embankments are typically 2 to 4 m amsl. If they are overtopped or breached the enclosed areas become part of a tidal-flat and flooded by saline water (Fig. 1c). In addition to monsoonal rains, driven by regional large-scale atmospheric circulations, there is the occurrence of tropical cyclones, with intense rainfalls and high wind speeds that affect coastal areas on an episodic basis (see ESM). These often induce storm surges inundating the low-lying coastal areas including polders. Such events can pollute surface water sources and near-surface stored drinking water resources with saline water and have a devastating effect on local communities.

Drinking water sources (groundwater, rainwater or surface water), and the means of collection and storage (tubewell, terracotta jar, pond etc.), which vary from area to area, are vulnerable to inundation. The major cause of inundation (i.e., ‘exposure’) along these coasts is cyclonic storm surge, and, very occasionally, by severe high-tide and/or tsunamis. The severity of inundation events is often determined by the elevation and geomorphology of the associated coastal segment. The impacts by inundation are exacerbated if the population density is high. Therefore, population density is used as a proxy for ‘sensitivity’. Furthermore, rainfall amount and fresh groundwater availability can be used as ‘resilience’ factor.

The analysis did not consider tsunamis, variation and duration of tidal amplitude, variation of river erosion, rate of relative sea level change, and variation of atmospheric pressure as variables along the coasts because of their scale dependency and interrelation with other variables (e.g., elevation and geomorphology) considered. Inclusion of these may improve the assessment in some specific areas, but at the expense of the regional goal. In contrast, most of these variables have limited influence on the drinking water vulnerability at the scale of current regional interest, and the relative risk would not change greatly because of many of their spatial similarity to elevation and geomorphology.

### Linkage of variables to vulnerability

**Fig.2:**
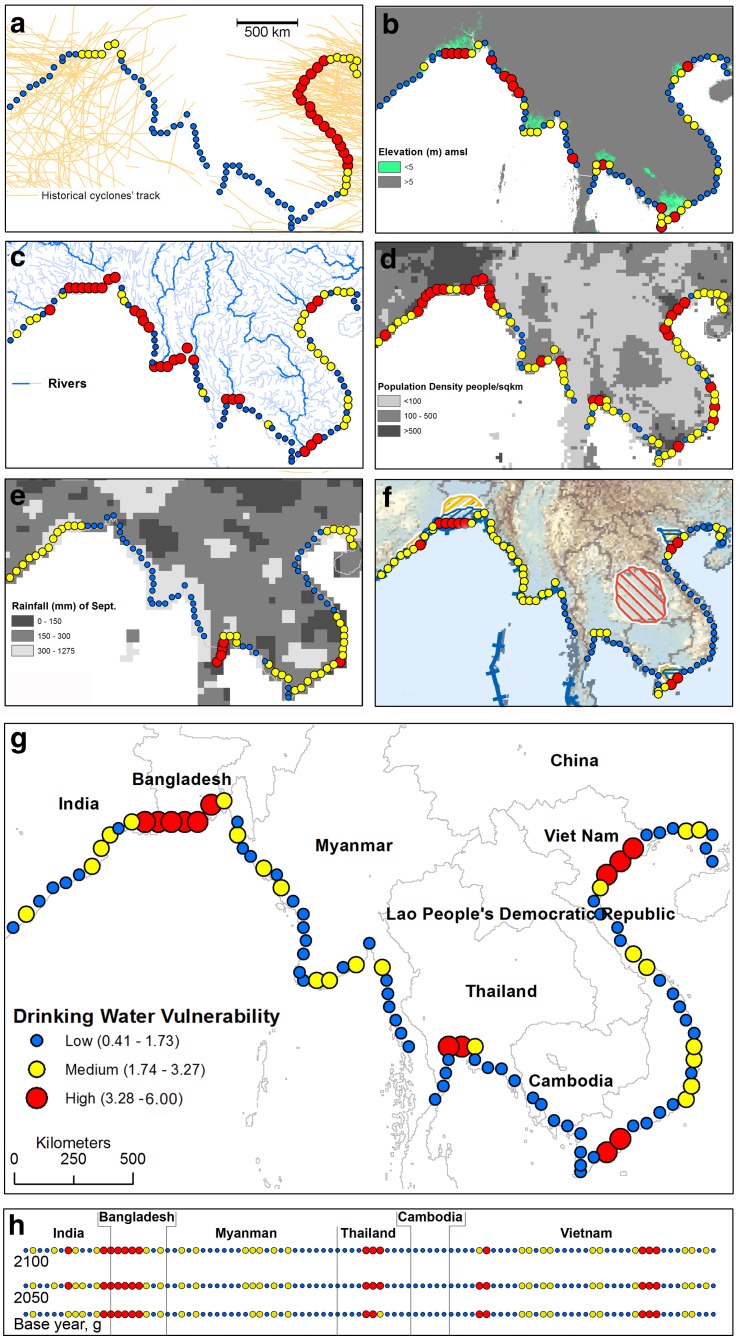
Vulnerability classes and combined vulnerability index. **a** Relative coastal vulnerability due to landfall of cyclone, based on incidence analysis of 162 years historical cyclonic tracks (1851–2012), **b** Vulnerability due to elevation differenecs in reference to mean sea level, **c** Geomorphological vulnerability classes, **d** Vulnerability due to population density, **e** Vulnerability due to rainfall variation, based on (110 years) average September rainfall, **f** Vulnerability associated with non-availability of fresh groundwater (the background map is taken from van Weert et al. (2009)), **g** combined relative vulnerability index along the coasts, and **h** combined relative vulnerability index for 2050 and 2100 in reference to 2012 considering the projected rainfall, sea-level rise, and population density. Note symbols for vulnerability classes (**a–h**) are the same of ‘g’, but associated values are CVI (Coastal Vulnerability Index) score only apply to ‘g and h’. For classification details, values, and sources of data see the text

The coastal area was divided into ½^0^ grid (ca. 50 × 50 km^2^) elements (Fig. 2) and a value for each of the 6 variables (Table 1) described above was retrieved from the gridded global surface models or maps, and integrated with available local information. The relevant element value is then classed as ‘low’, ‘moderate’ or ‘highly vulnerable’ as explained below.

**Table 1:** Vulnerability classes and indexing

**Indicators**	**Factor**	**Vulnerability classification**			
		**Definition**	**Low (1)**	**Moderate (2)**	**High (3)**
Cyclones	Exposure	Number (#) of category 1 to 5 cyclone landfall between 1851 and 2012. Higher # of cyclones, higher vulnerability.	<5	5–10	>10
Elevation (m, amsl*)	Exposure	How high an area is relative to sea-level. Higher elevation, lower vulnerability.	≥5	>2– < 5	≤2
Geomorphology	Exposure	Nature of coastal landforms: delta plain, sandy beach, and cliff. Flat landform, high vulnerable.	Beach with cliff	Beach with river mouth	Delta plain with river mouth
Population(people/km^2^)	Sensitivity	Number of people living per km^2^. Higher no. of people, higher vulnerability.	<100	100–500	>500
Rainfall (mm)	Resilience^ϒ^	Amount of September average rainfall. Higher the rainfall, lower the vulnerability	>300	150–300	<150
Groundwater	Resilience^ϒ^	Availability of fresh groundwater. Higher availability, lower vulnerability.	Fresh/less saline groundwater	Saline shallow groundwater	Saline shallow and deep groundwater

*amsl = above mean sea level

^ϒ^could be considered as exposure too but result would not change

#### Tropical cyclone

A global database of tropical cyclone tracks recorded between 1851 and 2012 (Knapp et al. 2010) was analysed. Only a few cyclones were uncategorised and these were excluded from the analysis. For simplicity, the analysis considered Category 1 (wind speed >119 km/h) cyclones and above, even though it is known that tropical storms (wind speed 63–117 km/h) and depressions (wind speed 0–62 km/h) can generate high-tides which can result in the occasional over topping of coastal polders. As the extent of a tropical cyclone is much wider than ½^0^, it was assumed that the cyclone had a spatial impact of ca 2^0^ (i.e., 4 elements). Depending on intensity and landfall frequency along a particular coastal segment, the vulnerability was ranked as ‘high’ (>10 cyclones over the recorded period), ‘moderate’ (between 5 and 10 cyclones) or ‘low’ (<5) to inundation due to storm surges (Fig. 2a).

#### Elevation

Elevation is the most important local factor affecting coastal inundation. The Shuttle Radar Topography Mission (SRTM) elevation dataset (EROS 2002) was used to assess this (Fig. 2b). Coasts were classified into ‘high’ (if elevation ≤2 m amsl), ‘moderate’ (between >2 and <5 m amsl), and ‘low’ vulnerability (≥ 5 m amsl) to inundation (Fig. 2b). As the mean tidal amplitude is around 2 m along theses coastline, this was used as a cut off value between high and moderate vulnerability classes. The heights of inundation due to cyclonic storm surge rarely exceed 5 m (Chapter 14 in SAARC 1992), and hence areas above 5 m amsl were considered safe from inundation.

#### Geomorphology

Coastal geomorphology here encompasses landforms, and indirectly processes such as waves, tides and currents acting to shape landform features (e.g., sandy beach, tidal flats, shelves and high cliff). Landform information extracted from Google Earth images (Google Earth 2013), coupled with river network data (Lehner et al. 2008), were used to assign a high vulnerability rank if a location is in a delta and near an estuary or river connected to a bay (Fig. 2c). Similarly beaches with a river emptying into sea were considered moderately vulnerable, and areas that featured only beaches were treated as low vulnerability zone.

#### Population density

Population density of 2010 was used as a measure of inundation impact, i.e., the higher the population the higher the impact. The socio-economic conditions were not considered, as these are fairly comparable among the rural coastal populations, even though separated by international boundaries. Population density for each square kilometre was retrieved from a global population model (SEDAC 2004), and used to classify each coastal element as high, medium, or low vulnerability, if the population densities were >500, between 100 and 500, and <100 people/km^2^ respectively (Fig. 2d).

#### Rainfall

The cumulative amount of rainfall at the end of the rainy season, particularly in September and October, is crucial in determining whether a major inundation event leads to drinking-water scarcity. It was estimated that around 300 mm rainfall would be sufficient to harvest 4000–6000 l (4–6 months’ supply for 5 people) of drinking water, based on a roof area of 15–20 m^2^. Rainfall data were retrieved from the National Oceanic & Atmospheric Administration (NOAA-ESRL 2011) gridded (0.5° grid resolution) global rainfall model (based on 1900 to 2010 observed data). Coastal elements (Fig. 2e) were classified into three categories with respect to average (for 110 years) September rainfall, accordingly as: low (>300 mm), medium (between 150 and 300 mm) and high (<150 mm) vulnerability.

#### Availability of fresh groundwater

The global map of salinity distribution in groundwater (van Weert et al. 2009) was combined with available local data (ESM section 3). The coasts were categorised into high, medium, and low vulnerability areas in terms of availability of fresh groundwater at shallow or deeper depths (Fig. 2f). A location was assigned as highly vulnerable when both shallow and deep groundwater are saline, while fresh groundwater at depth overlain by saline groundwater at shallower depths was defined as moderately vulnerable, and fresh groundwater at shallow depths as low vulnerability.

### Integrated vulnerability index and mapping

A summary of the individual vulnerability index of the 6 risk variables is given in Table 1. These can be combined to obtain an overall index of drinking-water vulnerability, where the coastal segment with high index values will tend to have more frequent tropical cyclones, low elevation, deltaic geomorphology, high population density, low rainfall, and saline groundwater (e.g., Daniels et al. 1992). Such an index may be used to identify areas where access to drinking-water sources is at risk. The Coastal Vulnerability Index (CVI), described by Daniels et al. (1992) and Gornitz et al. (1997) and adopted in McLaughlin and Cooper (2010), provides a simple numerical basis for ranking sections of a coastline in terms of water resource susceptibility to increased salinity, and helps identify regions where risks are relatively high. For ease of use, a semi-quantitative scale of 1–3, where 1 indicates low vulnerability, while 3 indicates high, was used.

Following the sensitivity analysis by Gornitz et al. (1997), the 6 key variables are combined into a single index using the square root of their product mean (Eq. 1) (see ESM Figs. S5 and S6). The assessment of vulnerability based on the equal contribution and interaction of the six variables may not be entirely reasonable, but giving different weighings to them is complicated and subjective. Therefore, for simplicity, and for combined relative vulnerability estimation, an equal weighting of unity was used.



Where different vulnerabilities are indicated by *a* = elevation, *b* = geomorphology, *c* = cyclone frequency, *d* = rainfall, *e* = groundwater availability, and *f* = population density.

## Results

### Variables for vulnerability assessment and mapping

#### Tropical cyclone

The analysis indicates that the Vietnam coast is most vulnerable along the Asian coasts, and the coastal zones of West Bengal of India and Bangladesh are moderately vulnerable. The number of historical landfalls of cyclones is highest along the Vietnam coast (>15 for Category-1 or stronger cyclones), followed by Bangladesh and the east coast of India (>7 Category-1 or stronger cyclones) (Fig. 2a).

#### Elevation

The classification indicates that deltaic and low-lying areas are moderately to highly vulnerable (Fig. 2b). The deltas have a total area of over 90,000 km^2^ below 5 m amsl elevation. The extent of the 5 m amsl elevation contour reaches >350 km inland for the Bengal and Mekong deltas. Furthermore, the width of the low-lying (i.e., <5 m amsl) area along the coast is >150 km in all these deltas, and is over 300 km in the Bengal delta. Away from these deltas elevation is steep and often abated by adjacent hilly topography, with occasional local and minor delta-like features present where small rivers meet the sea. The most prominent (ca. 200 km × 35 km) of these low-lying areas is located in Rakhine State, Myanmar, in between the Bengal and Irrawaddy deltas.

#### Geomorphology

The coastal region investigated has a range of depositional landforms: beaches, barrier bars, beach ridges, bay mouth bars, intertidal flats; where tidal flats and beaches are dominant landforms. Tidal flats occupy the deltas and low-lying areas and rest of the coast is sandy beaches, where tidal inundation is limited to a narrow zone.

Geomorphological analysis indicates that the deltaic coasts are highly vulnerable (Fig. 2c) and are characterised by a high number of rivers and tidal creeks connected to the sea/embayment and low elevation. Bengal and Irrawaddy deltas have wider tidal creek network coupled with large tidal flats making these more prone to wider area inundations from a storm event.

#### Population density

The area is one of the most densely populated coastal regions of the world with deltas being highly populated, and centres for agricultural production. The analysis shows that the deltaic coasts are more vulnerable than the other parts (except southeast Vietnam coast, where population density is also high) of the SSE Asian coastline (Fig. 2d). Among the deltas, both Bengal and Red River have >1000 people/km^2^, while other vulnerable segments have population densities <1000 and generally around 500 people/km^2^.

#### Amount of rainfall

The mean (110 years) September rainfall is 350 ± 144 mm with most places exceeding 200 mm, only in a few places rainfall is <150 mm, the lowest being 127 mm. Average September rainfall is less than 300 mm in southern Vietnam and in coastal Thailand, and also in western parts of the Bengal delta. This makes the eastern region, i.e., Vietnam-Thailand coasts, relatively more vulnerable than the western region i.e., Myanmar-Bangladesh coasts (Fig. 2e).

#### Availability of groundwater

In terms of fresh groundwater availability, eastern (Vietnam-Thailand) coasts are less vulnerable than the western coasts (Indian-Bangladesh-Myanmar) (Fig. 2f). The occurrence and use of fresh groundwater vary within small segments of the coasts. For instance, along the eastern part of the Bangladesh coast, >90 % of the population rely on deep fresh groundwater (e.g., Ravenscroft et al. 2009) for drinking water, while in the western part a similar fraction relies on rainwater and other surface water sources (Islam et al. 2013) to meet drinking water demand due to the lack of fresh groundwater. Similar situations have also been reported in the Mekong delta (Delta Alliance 2011), and may also exist in other coastal parts. Shallow groundwater along the Vietnam-Thailand coast is relatively fresh and often developed by hand-dug wells (Anderson 1978).

### Integrated vulnerability indexing and mapping

Exploring all possible combinations for the six variables according to Eq. 1 (ESM Fig. S5) allows the range of possible outcomes to be compared to other calculation methods (e.g., mean) (ESM Fig. S6). It shows that CVI values range between 0.41 and 11.02. As expected, there are fewer high values compared to lower ones. In our current analysis the maximum CVI is 6.00, and only 5.1 % (*n* = 98) have CVI values >5.00 (ESM Fig S6). Finally, CVI values are classified into 3 different groups (low, medium, and high) using 1.73 (3 lows + 1 medium + 2 highs) and 3.27 (all medium) as limits (ESM Fig. S5). Although these thresholds are, to a certain extent, arbitrary, they allow a clearer identification of the relative vulnerabilities of different elements, and indicate which are the most vulnerable. The integrated CVI indicates that deltas are the most vulnerable (Fig. 2g).

## Discussion

### Relative vulnerabilities and key areas

The relative vulnerability assessment indicates that vulnerability of drinking water to salinisation is highest along the deltaic segments, and that this is primarily due to low coastal elevation, and the associated geomorphology. It is estimated that the total length of highly vulnerable elements is around 900 km, with more than 1500 km being moderately vulnerable (Fig. 2g). This supports previous findings by Saito et al. (2007), who identified the SSE Asian coasts, particularly the deltaic segments, as one of the environments most vulnerable to future global changes.

The integrated vulnerability index indicates that the Bengal and Red River deltas are particularly vulnerable, and also some areas in the Mekong and Chao Phraya deltas (Fig. 2g). The wide shallow water shelves adjacent to these deltaic segments (Voris 2000) have an amplifying impact on inundation (Resio and Westerink 2008). In addition, the concave geometry of the deltaic coasts focuses the surge into smaller areas, resulting in greater depths of inundation, and the wider river mouths allow the surge water to flow more easily and quickly through the landscape, which can be as far as 30 to 50 km inland (Al-Salek 1998). These processes cause a high risk of salinisation of drinking water in more than 35,000 km^2^ near-inland and coastal areas of these deltas. Consequently, it is estimated that more than 25 million people, within 30 km from the coast, are living in highly vulnerable areas, while another ca. 30 million people are living in moderately vulnerable areas.

### Impacts of climate change

Global sea levels have risen between 17 to 21 cm during the period 1901 to 2010, and are predicted to rise a further 26 cm to 98 cm by 2100 (Chapter 13, (IPCC 2014; Nicholls and Mimura 1998)). Further exacerbating the hazardous nature of some of the natural processes described above. As an example, the rate of relative sea level rise in the Bay of Bengal (between 1977 and 1998) is 4.0 to 7.8 mm/year (Singh 2002), which is much higher than the global average of 1.8 ± 0.3 mm/yr. (Church et al. 2004). The rate of sea level rise is generally higher along the India–Bangladesh–Myanmar coast (ca. > 4 mm/yr) compared to the Thailand–Vietnamin coast (ca. >1.5 mm/yr) (Church et al. 2004). However, within these two respective coastlines the rate is higher along the deltaic segments. The coastal region of SSE Asia is vulnerable to sea level rise, and could lose around 10 % of the land area by 2100 (Nicholls and Mimura 1998). However, Nicholls and Mimura (1998) did not consider reclaimed land contained in polders, the height of which, it is assumed, will be raised in order to protect this land. This means that the current area of over 25,000 km^2^ in the Asian mega deltas below 2 m amsl, could increase by 60 % under projected 21st century sea level rise (i.e., 1 m). This would transform the SSE Asian coasts into an increasingly hazardous region. Furthermore, shoreline recession combined with sea level rise (SLR) is likely to accelerate coastal erosion (Ericson et al. 2006), and may, therefore, lead to some loss of land as well as frequent failure (i.e., breaching) of coastal polders, which would increase the risk of drinking water salinisation.

Deltas, therefore, remain as persistent vulnerable zones throughout the 21st century (Fig. 2h) and it is expected that, within deltaic segments, the impact of climate change will vary. A study along the Bangladesh coast indicates that storm surges occuring as a result of a 2.0 °C sea surface temperature (SST) rise and a 0.3 m SLR, would increase the flood risk area by 15 % of the present area, and the depth of flooding could increase by as much as 23 % for locations within 20 km from the coastline (Karim and Mimura 2008). However, historical data on storm frequencies on a global scale are inconclusive (Hijioka et al. 2014). By contrast, it is considered that, due to climate change in 2050 and 2100, respectively, population density might be 40 % and 35 % more than 2010 (UN 2004), elevation (due to sea level rise) will be 0.2 and 0.6 m less than current elevation (Chapter 13, IPCC 2014), rainfall could increase by 5 % and 10 % (Hijioka et al. 2014), whilst other variables (e.g. tropical cyclone, geomorphology, fresh groundwater availability) probably will not change. Thus, the assessment of vulnerability for 2050 and 2100 indicates no significant change at the scale of investigation and deltas remain the most vulnerable segments (Fig. 2h).

### Economic activities and coastal hazards

Economic activities in coastal and upstream areas have direct impact on coastal hazards, in particular the construction of dams or major abstractions of water for irrigation (Shearman et al. 2013)Among the considered deltas, the Irrawaddy is less influenced by upstream damming and withdrawal of water, and has, therefore, been maintaining a balance between sedimentation and subsidence. This delta also rarely experiences tropical cyclones, which possibly encouraged the destruction of around 80 % of its coastal mangrove-forest to be replaced by rice paddies and shrimp farms. Had more mangroves been left to survive, the impact of the storm surge by the deadly Cyclone Nargis might have been lessened (Hedley et al. 2010). The upstream withdrawal of water in Asian rivers has increased substantially since the 1950s and has already resulted in increased river salinity and landward intrusion of saline water in the Bengal delta (Mondal et al. 2013), and other Asian deltas (Saito et al. 2007). In the dry season the salinity front moves up to 150 km inland in Bangladesh (Mondal et al. 2013) and about 70 km in the Mekong river delta (Noh et al. 2013).

Gopalakrishnan (2013) critically showed that, generally, when water related disasters are considered, water resources are rarely considered in terms of management policy-framework. Post disaster availability of potable water becomes very critical, as aid cannot reach the sites because of the failure of road networks. During an inundation event (e.g. at a scale of that shown in Fig. 1c) many drinking water sources could be polluted by saline water. When the flood water recedes, (drinking water) ponds can contain entrapped saline water (if not pumped out) and it can take around 5 to 7 years for salinity to return to its background value through dilution by rainwater (Ortega 2009). To identify key vulnerable areas at the local scale, mapping should be done with more detailed information, particularly previous inundation history, the condition and elevation of polders, the quality of local infrastructure, and also any potential influences of upstream and other economic activities.

### Drinking water salinity and health

Drinking water salinity poses a threat to human health (ESM Fig. S7). In some parts of coastal SSE Asia, drinking water is provided by perennial fresh groundwater, where adverse effects on heath are not largely a concern. In contrast, where groundwater is too saline people rely on harvested rainwater. Due to varied methods of collection, rainwater often has added components (e.g. dust, insects and bits of vegetation etc.), and storage jars can become the breeding ground for mosquito (Tran et al. 2010), and pathogens or insect ova (Wilbers et al. 2013) leading to outbreaks of diarrheal diseases and dengue (Fig. S7). Similarly, communal ponds may also lead to diarrheal diseases if the water is not treated for pathogens.

However, consumption of sodium through drinking saline water forms a significant part of the total sodium intake in these areas (Hoque and Butler 2016). Research in a deltaic area in southwest Bangladesh revealed that, towards the end of the dry season, half of the population - relying on ponds - were consuming at least 25 % of their total sodium intake unknowingly through drinking water: 1 out of 3 even consumed 50 % or more of their total sodium intake only through drinking water (P. Scheelbeek, unpublished data). It is very likely that the inhabitants in these settings are chronically exposed to excessive sodium intake because drinking water sodium is ancillary to food derived sodium. The association between excessive sodium intake and increased risk of hypertension is widely known (e.g., WHO 2012). Furthermore, Khan et al. (2014) have found an association between drinking water sodium and preeaclampsia (a condition in pregnant women characterised by high blood pressure) in a salinity prone area in southern Bangladesh. It has been estimated that more than 7 million people residing in highly vulnerable coastal segments may have hypertension/high blood-pressure attributable to drinking water salinity (P. Scheelbeek, unpublished data).

Health risks from water-borne diseases are widely known, but our knowledge of hypertension associated with saline drinking water is not widespread, and was not even mentioned in the recent IPCC report (Chapter 11, IPCC 2014). Moreover, the WHO does not have a guideline for maximum sodium levels in drinking water, claiming the contribution from drinking-water to daily intake is [always] relatively small (WHO 2011). However, exposure to saline drinking water is likely to be prevalent along the deltaic coast of SSE Asia (Hoque and Butler 2016). Although the episodic and seasonal increase of the prevalence of hypertension along these coastal areas is not yet quantified, it is likely to be significant.

### Alternatives for Adaption

People have been living along these coasts for centuries, and surviving against natural hazards by adapting to the changing climatic condition. Mortality and morbidity associated with cyclones have been reduced significantly with the advancement of understanding, and the provision of warning systems, disaster shelters, and rescue and evacuation procedures (Shultz et al. 2005). Although, emergency efforts supply interim drinking water (e.g., bottle water, portable water plants etc.), it rarely look into longer term solutions against salinisation of drinking water induced by inundation. Adaptation to these would require modification of the natural environment, change in practice and engineering. Here we outline some adaptation alternatives to ensure the protection of drinking water (sources, means of collection and storage) against inundation to safeguard year-round supply.

#### Natural environment modification

Coastal inhabitants, particularly those living in deltas within the embanked areas, reclaimed from the regular tidal-regime. Communities often reside near to embankments for convenience of communication. Overtopping of these embankments can potentially contaminate communal drinking water ponds within the polder. Increasing the height and regular maintenance of the embankments will reduce the risk of overtopping and/or breaching of embankments and the related salinisation of drinking water.

#### Changes in practice and engineering solutions

Traditionally, rainwater is harvested in terracotta jars stored on the ground. As a change of practice these jars should be placed in an elevated location within the house to avoid the salinisation of the stored water from inundation events. Large-scale communal rainwater harvesting would also help to reduce inundation risks if these are designed with the top of the tank above the typical level of inundation (ESM Fig. S8). Fresh deep groundwater (>150 m) occurs in patches and heterogeneously along the coastal region, is a source of perennial supply, and identifying these freshwater zones could be an alternative water resource. However, the hand tubewell used to tap these water in rural areas are often completed on the ground making this resource worthless as saline water overtopping and/or breaching of embankments and drown the tubewell. Raised (above the frequent high tide level) concrete platforms should be fitted with every tubewell in coastal areas to ensure year-round supply of water including during an inundation event.

The use of aquifer storage and recovery (ASR) by creating a freshwater bubble in saline aquifers, which is later recovered after some storage, is relatively new (Ward et al. 2009) In Bangladesh several ASR units, where excess rain water and fresh pond water is injected into the subsurface, have been in operation for the last 3 years (Sultana et al. 2015), and each unit is supporting a small coastal community (ca. 50 households) throughout the dry season (ESM Fig. S9). An exploratory analysis of all required elements (low hydraulic gradient, confined or semi-confined nature of shallow aquifer, and adequate amount of rainfall) indicates that a vast area in Asian deltas has potential for implementation of ASR (ESM Fig. S10), and can be an alternative climate-proof freshwater source, particularly during the dry season.

Desalinisation could also be an option to produce fresh water from saline or brackish water but the cost and required supply of electricity is a major problem in these resource poor-settings. However, desalinisation by solar distillation, using solar heat to evaporate pure water from saline water (Sampathkumar et al. 2010) could be an alternative during the dry season.

## Summary

Drinking water sources along SSE Asian coastal plains are at risk from salinisation due to episodic storm surges. These risks are likely to increase over the coming century due to rising sea levels and more frequent and/or intense tropical cyclone activity. Prioritisation of those areas most at risk is essential in order to prevent serious health impacts in the short term from the immediate impacts of such events and over the longer term from ingestion of excessive salt. A coastal vulnerability index has therefore been developed incorporating six variables, associated with historic cyclone activity, ground elevation, coastal geomorphology, population density and availability of fresh water (rain water and/or groundwater), in order to derive a relative vulnerability map. This shows the most vulnerable coastal zones to be the Bengal and Red River deltas, and also some areas in the Mekong and Chao Phraya deltas. This means that the many millions of people living in these areas are at risk of being exposed to high levels of salinity and the health impacts associated with excessive salt intake, which could increase further under climate change. A variety of adaptations (either from practical protection measures) or novel alternative drinking sources (such as aquifer storage and recovery) are proposed in order reduce these risks.

## Electronic Supplementary Material

ESM 1 (PDF 3.66 mb)
