# Supplementary material for: Drinking water vulnerability to climate change and alternatives for adaptation in coastal South and South East Asia
Source: Clim Change. 2016 Feb 24;136:247–63. doi: 10.1007/s10584-016-1617-1 (PMC4944792; doi:10.1007/s10584-016-1617-1)
Supplement: Supplementary file 1 — (PDF 3.66 mb) [file 10584_2016_1617_MOESM1_ESM.pdf]

Electronic Supplemental Material

Drinking water vulnerability to climate change and alternatives for adaptation in coastal South and South East Asia

M. A. Hoque[1*], P. F. D. Scheelbeek[2], P. Vineis[2], A. Khan[2], K. M. Ahmed[3], A. P. Butler[1]

[1]Department of Civil and Environmental Engineering, Imperial College London, UK

[2]MRC-HPA Centre for Environment and Health, Department of Epidemiology and Biostatistics, Faculty of Medicine, Imperial College London, UK

[3]Department of Geology, University of Dhaka, Bangladesh

*E-mail: m.hoque@imperial.ac.uk; Tel: + 44 (0)20 759 46074

1. Climates and tropical cyclones

The dominant climatic systems along these coastlines are the Asian monsoons, in particular, the Indo-China Peninsula, South China Sea, South Asian (Indian), East Asian (south China, lower Yangtze River and Japan) and Northeast Asian (north China and Korea). Their onset periods take place in late April – mid-May, mid-May, mid-May – late-July, mid-May – late-June and late-June – mid-July, respectively (Qian and Lee, 2000). The area has one of the highest amounts of rainfall in the world. Analyses of 110 years annual rainfall data (NOAA-ESRL, 2011; Schneider et al., 2011) show that their average annual rainfalls range between 1350 mm/year (Chao Phraya delta) and 2860 mm/year (Irrawaddy delta) (Fig. S1), with 85% of the rainfall occurring between May and October.

The monsoon, driven by regional large scale atmospheric circulations, also allows formation of tropical cyclones, which effect coastal areas on an episodic basis. In general, more intense cyclonic winds generally produce a higher storm surge height, given all other variables (such as the cyclone's areal extent, local bathymetry, local topography, cyclone's forward speed, and the angle at which the cyclone approaches the coast) being equal (e.g., McIvor et al., 2012). However, these additional factors often play a very significant role in how a surge impacts along the coast. For instance, cyclone Sidr (land fall on November 15, 2007), which was a Category 4-equivalent cyclone (according to the Saffir–Simpson Hurricane Scale NOAA, 2005), with a sustained wind speed of 260 km/h, inundated a significant area in coastal Bangladesh (e.g., Islam et al., 2011). Whereas, cyclone Nargis, which was Category 1-equivalent cyclone (landfall in Irrawaddy delta of Myanmar on May 2, 2008), inundated a larger area and caused 140,000 fatalities (Fritz et al., 2009; Tripartite Core Group, 2008). One reason for its extensive impact was that the surge hit during high tide, greatly increasing its intensity. Furthermore, cyclone Aila (landfall May 21, 2009), which was a Category 2-equivalent cyclone with a sustained wind speed of 110 km/h, inundated an area greater than Sidr in Bangladesh and West Bengal (Mallick et al., 2011) (Fig. S2). Storm surges associated with these cyclones overtopped polders (earthen embankments

placed around the coastal tidal rivers) and salinised drinking water ponds, and also drowned household terracotta jars (<1 meter high, traditionally kept on the ground) that store harvested rainwater.

Cyclones normally make landfall between April and November i.e., throughout the rainy season. Cyclone Sidr made landfall in November, while Nargis and Aila in May. Cyclonic frequency and landfall timing also vary along the coasts. For example, in the earlier part of rainy season (June-July), cyclones hit the northern part of Vietnam coast and migrate southward by the end of the season (August-September) (www.nlcap.net, accessed on 14 Nov 2013). The frequency of cyclones in the Indian Ocean is higher at the end of rainy season, during August-September. Siddiki et al (2012) analysed 100 years (1908–2008) historical cyclones and depressions generated in the Bay of Bengal and found that these are frequent in August (>20%) putting September as a next frequent month for storms. However, landfall of cyclones in the region is equally dominant in May and November, and some of the deadliest cyclones hit the coasts during these two months (Alam et al., 2003). Cyclonic depressions formed in the sea often do not make landfall but generate high-tide causing occasional over topping of coastal polders. The tropical cyclones and cyclonic depressions could, in turn, lead to an increase in rainfall amounts (Zheng et al., 2013), and therefore more inland flooding during storm events.

The coastal region of current interest is around 6000 km long, and characterised by different kind of depositional environments: beach, barrier bars, beach ridges, bay mouth bar, intertidal flats. Among those tidal flats and beaches are prominent. Tidal flats occupy the deltas and low-lying areas, undergo semi-diurnal tide where elevation of the land is <2 m amsl. On the contrary, beaches, dominantly sandy, adjust quickly to changes in wave and tidal energy, and tidal inundation is narrow aerially. The shallow water shelves is wider adjacent to the tidal flats i.e., in deltaic region compare to the beaches along these coasts (Voris, 2000), has amplifying impact on inundation (Murty et al., 1986; Resio and Westerink, 2008). Concave geometry of coasts focus the surge into a smaller area, resulting in higher water levels, and larger river mouths allow the surge to flow more easily and quickly through the landscape (Al-Salek, 1998; Johns and Ali, 1980; McIvor et al., 2012).

The said coasts are also vulnerable to tsunami and can inundate the coastal regions with sea water. One major difference, however, is that, depending on its magnitude, the impact area of a tsunami could be much wider than a storm surge from a tropical cyclone, and could, therefore, inundate areas that are normally safe from storm surges (Løvholt et al., 2012). For example, the tsunami of 26 December 2004 impacted the entire coast of the north Indian ocean (ibid), while the impact from storm-surges is restricted to just part of the coast.

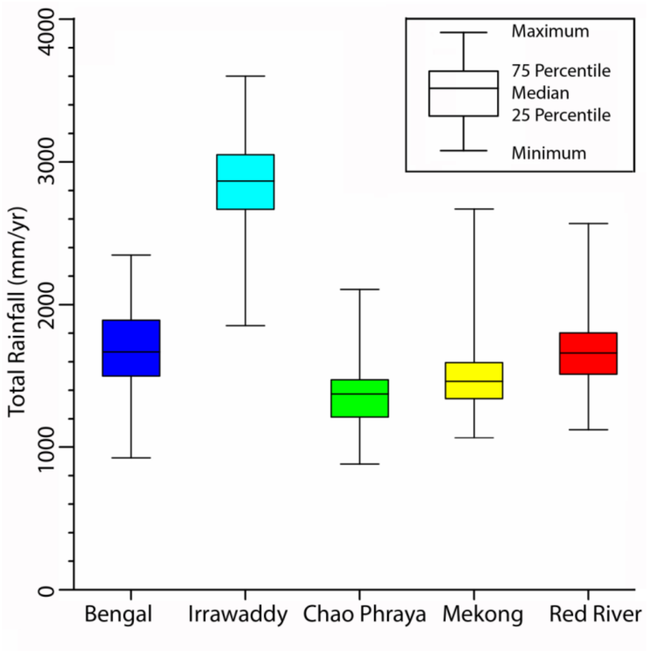

Figure S1: Summary of 110 years annual total rainfall in the various deltas along SSE Asian coastline. Data was retrieved for the black dot locations in Fig. 1a, see text for details.

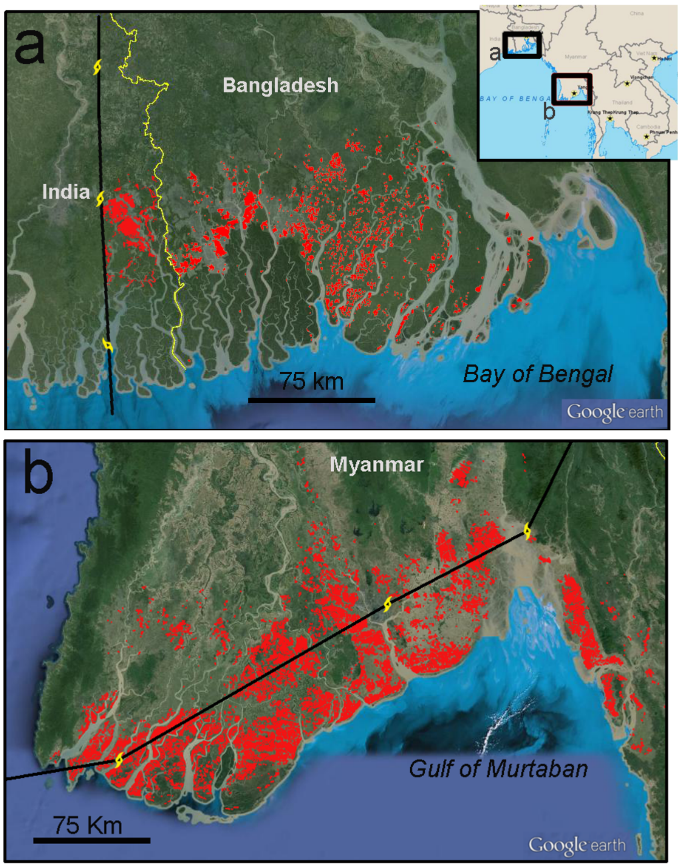

Figure S2: Extent of inundation caused by tropical storm surges: a) in West Bengal, India and southern Bangladesh during the Cyclone Aila (21st of May 2009), b) in Irrawaddy delta during the Cyclone Nargis (2 May 2008), based on 6th May 2008 satellite image. Cyclone tracks are indicated by black lines. Inundation areas are retrieved from www.ithacaweb.org/maps/, accessed on 01 Nov 2013, and overlaid on Google Earth images.

2. Drinking water crisis created Aila and Nargis

Cyclone Aila: In coastal Bangladesh, particularly southwest region, people rely on multiple sources of water as mentioned in the main script. Women and children are generally responsible for collecting water, and due to scarcity of freshwater often they spend 2.5 hours every day to fetch fresh water (Mallick et al., 2011). After cyclone Aila water scarcity increased significantly as most of the pond sand filters become useless because of contamination of the source-pond by the inundation (Fig. S2a) of saline water. As the embankments remain not-fixed, breached due to the cyclone, more than a year people had to live on the roads, which lead to poor sanitation in the regions. As a result, water-borne diseases broke out like: dysentery, cholera, diarrheal diseases, skin diseases and fever. Since the area remained water logged, women and children had to spend more time in collecting water, and this also led to drop-out from the school. Most people relied on limited relief provided by the government and NGOs, and drinking water supply was not adequate leading often relying on conventional sources, high in salt and/or pathogens.

Cyclone Nargis: Reliance on multiple sources of water for collecting drinking water is also common in Nargis affected areas in Irrawaddy delta. During the rainy season, rain water harvesting provided 30% of the water supply for communities. However, with the beginning of the dry season (November to April), there is an increased dependence on other water sources including surface water. The extensive contamination of surface water bodies by the cyclone induced storm surges (Fig. S2 b) diminishes the water availability across and within communities over the dry season. In addition, disaster migrant from other areas of the delta (that were hardest hit by the Cyclone) had placed increased demands on existing reserves of potable water. Water treatment in the hardest hit areas were low, only 5% people used chlorination, while around 35% people used boiling or sand filters to treat water, but rest did not used any treatment (Tripartite Core Group, 2008). As salinity could not be removed through any of these treatment people left with no choice but drinking relatively saline water where fresh water was not available.

3. Hydrogeology and Aquifer system

Although, the geology of the area extends from Precambrian to Recent time, and varies from fluvio-deltaic plains of the major rivers along the coasts to hard rock terrains and mountainous regions away from the coasts (Lee and Lawver, 1995) the exploitable aquifer systems have been hosted by the Quaternary sediments largely. Also, the current landform, particularly in deltas, has been determined during the Late Quaternary by the eustatically induced geological modifications (Goodbred and Kuehl, 2000; Saito et al., 2007; Ta et al., 2002; Tanabe et al., 2006; Voris, 2000). The Holocene sediments overly older sediments of a similar origin from previous eustatic cycles form the multi-aquifer framework and meeting the demand of water for drinking and irrigations.

Groundwater resources in coastal region at shallower depths are pervasively impacted by natural salinity, and in some areas over-exploitation aggravate the situation (IPCC, 2007; van Weert et al., 2009) but in some areas deeper groundwater is fresh (Delta Alliance, 2011; Ravenscroft et al., 2013). The development of the deep groundwater is expensive and often development of such is not possible at individual level. Shallow, particularly at very shallow, groundwater along the Vietnam-Thailand coast is relatively fresh and often developed by hand-dig well (Anderson, 1978). The global map of salinity distribution in groundwater (van Weert et al., 2009)

combined with available local data (Buschmann et al., 2008; Delta Alliance, 2011; DPHE/DANIDA, 2001; Ravenscroft et al., 2013) provide a better understanding of the groundwater salinity distribution along the Asian coast as showed in main script (Fig. 2).

4. Climate change

Effects from global climatic change in SSE Asia are believed to be occurring but the mechanisms are not fully understood (Chotamonsak et al., 2011). It is known that sea-level rise and increases in sea-surface temperature, large tidal variations, possible increase in frequencies and intensities of tropical cyclones, coupled with the probable increase of regional rainfall, will potentially turn the coastal regions increasingly vulnerable. The mean temperature over a broad region encompassing Bangladesh has increased by about 0.5^0C over the past century (Karim and Mimura, 2008). Previous modelling shows that the mean temperature in the coastal region of SE Asia (Bangladesh, southern Myanmar, central and southern Thailand, Cambodia, southern Vietnam) will increase by $0.0\text{-}1.0^0$C by 2050s in reference to the base year 1990s, and mean sea-surface temperature (SST) will increased by $1.0 – 2.0^0$C (Chotamonsak et al., 2011) or more (Hijioka et al., 2014). All modelling studies indicate that the temperature increase is greater in winter compared to summer.

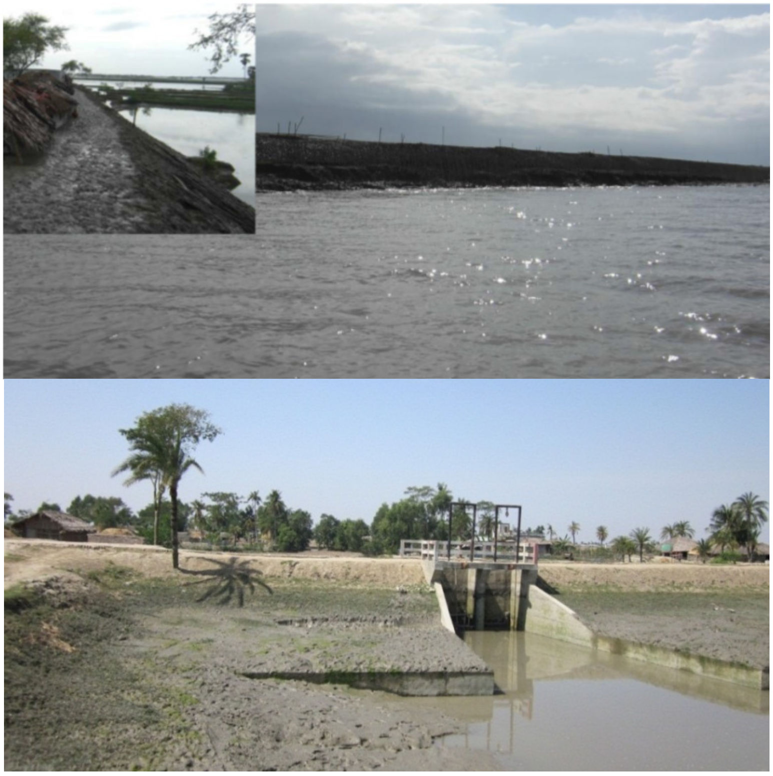

Figure S3: Photograph of a typical polder, earthen embankment, above, source: (Ortega, 2009), and a sluice gate structure of a polder in Dacope - a coastal area in Bangladesh. These are typical of other Asian deltas. In Bangladesh alone, 123 polders, including 49 sea-facing polders, were constructed in the 1960s to protect low-lying coastal areas from tidal floods and saline intrusion (The World Bank Group, 2010).

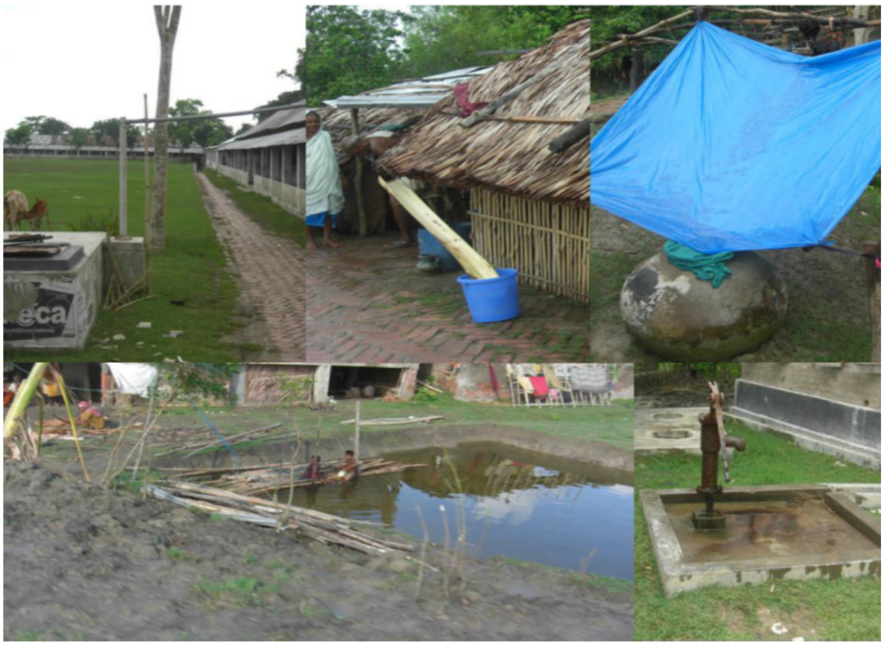

Figure S4: Illustration of drinking water sources in coastal areas. Source: (Ortega, 2009).

Numerical values of vulnerability indices	Variables						Total score	
	a	b	c	d	e	f	From eq. 1	Mean
	3	3	3	3	3	3	11.02	3.00
	2	3	3	3	3	3	9.00	2.83
	2	2	3	3	3	3	7.35	2.67
	1	3	3	3	3	3	6.36	2.67
	2	2	2	3	3	3	6.00	2.50
	1	2	3	3	3	3	5.20	2.50
	2	2	2	2	3	3	4.90	2.33
	1	2	2	3	3	3	4.24	2.33
	2	2	2	2	2	3	4.00	2.17
	1	1	3	3	3	3	3.67	2.33
	1	2	2	2	3	3	3.46	2.17
	2	2	2	2	2	2	3.27	2.00
	1	2	2	2	2	3	2.83	2.00
	1	1	2	2	3	3	2.45	2.00
	1	2	2	2	2	2	2.31	1.83
	1	1	1	3	3	3	2.12	2.00
	1	1	2	2	2	3	2.00	1.83
	1	1	1	2	3	3	1.73	1.83
	1	1	2	2	2	2	1.63	1.67
	1	1	1	2	2	3	1.41	1.67
	1	1	1	1	3	3	1.22	1.67
	1	1	1	2	2	2	1.15	1.50
	1	1	1	1	2	3	1.00	1.50
	1	1	1	1	2	2	0.82	1.33
	1	1	1	1	1	3	0.71	1.33
	1	1	1	1	1	2	0.58	1.17
	1	1	1	1	1	1	0.41	1.00

Figure S5: Possible combinations of 6 vulnerability indices and their total score using the equitation in the main text, and average of the vulnerability values. We used 1.73 and 3.27 as a cut of values for upper limit for low and medium vulnerability.

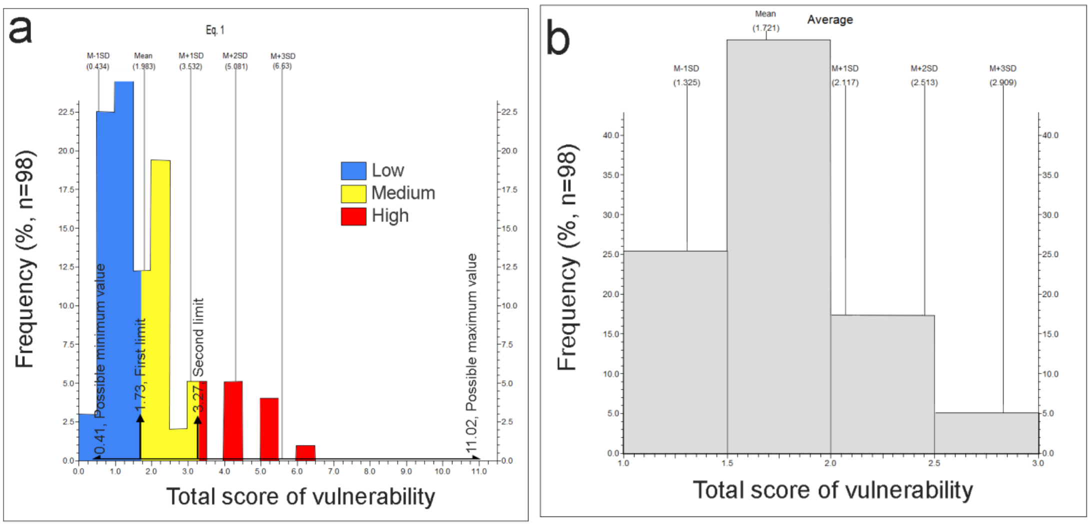

Figure S6: Frequency histogram (a. based on equation 1 in the text; b. Mean of the indices values) of our data indicate that the upper possible values are absent in our data, and also less frequent. We used 1.73 and 3.27 as a cut of values for upper limit for low and medium vulnerability.

5. *Salinity and Health*

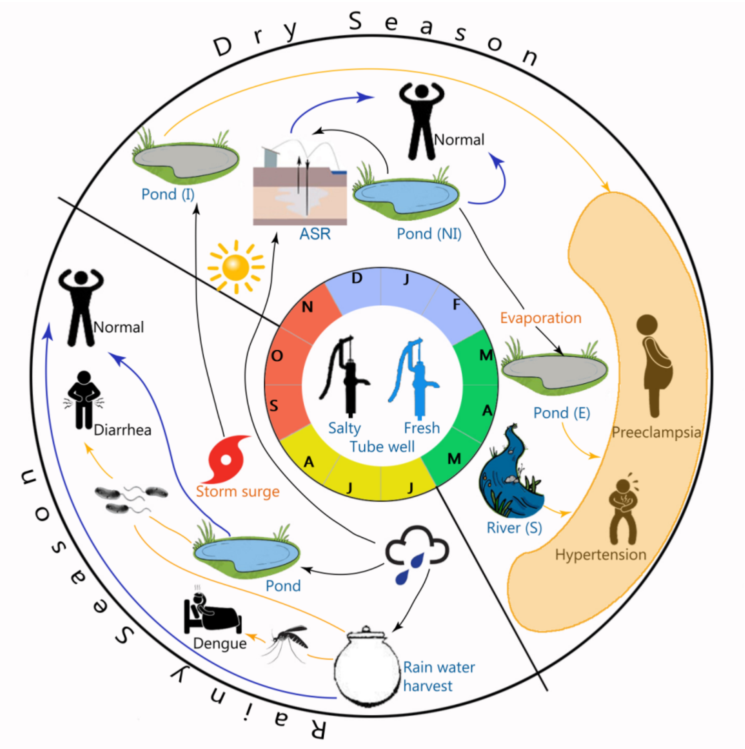

Figure S7: The reliance of multiple sources and the interaction of hydrology and health in coastal settings. Problem to heath is not a concern where drinking water is met by perennial fresh groundwater (blue tubewell in the centre). But people rely on rainwater, pond water and other sources, where groundwater is too saline (black tubewell in the centre), causes various health problems including vector-bone diseases. Episodic cyclonic storm-surges (in the months of August-September of the year) can cause salinisation of drinking water ponds. However, even if not impacted (NI) these can become increasingly saline due to evaporative losses towards the end of dry-season. Drinking saline water causes hypertension and preeclampsia to the consumers; prevalence is higher at the end of dry season (Khan et al., 2011). Rainwater, aquifer storage and recovery (ASR, see Figure S9) and NI ponds all have low levels of salinity and therefore mitigate against saline health impacts. See text for further details.

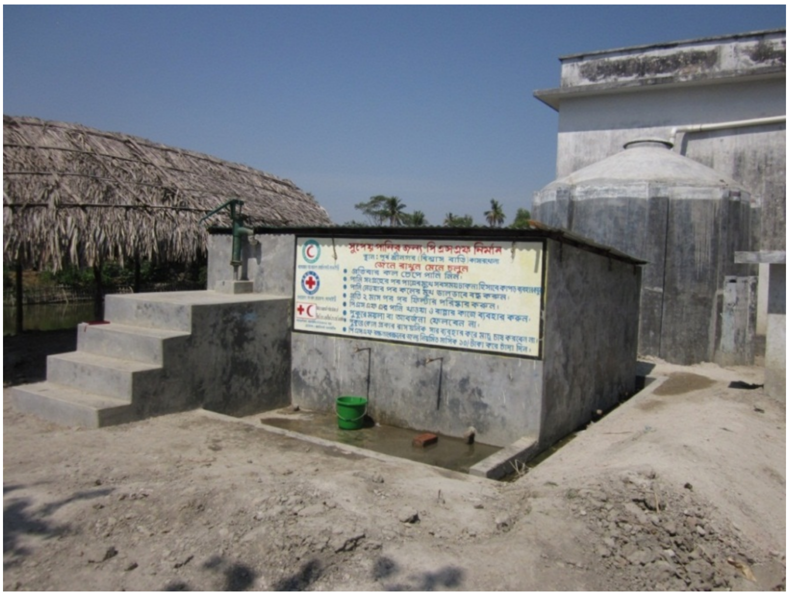

Figure S8: Community base rainwater harvesting and pond sand filter (PSF).

6. *Aquifer Storage and Recovery (ASR)*

The ASR is an old technique, which has been in use for centuries in Europe (Farnsworth and Hering, 2011). The use of ASR to source freshwater by creating a freshwater bubble in saline aquifer is relatively new (Miotliński et al., 2014; Ward et al., 2009). In this case rainwater is infiltrated during the rainy season into a saline coastal aquifer, which creates a freshwater bubble by pushing the saline water away, to be used in the following dry season. In Bangladesh several ASR units have been in operation for the last 3 years (Fig. S9) (Ahmed et al., 2010; Barker, 2013; Hasan, 2012; Sultana et al., 2015), and each unit is supporting a small coastal community (ca. 50 households) throughout the dry season. Modelling study by Barker (2013) shows that the current design of ASR in Bangladesh needs to be improved by increasing the height of the infiltration system (currently ca. 1 m above ground level), and putting the abstraction well on raised platform to avoid inundation induced infiltration of saline water.

All the geological factors (low hydraulic gradient, confined or semi-confined nature of shallow aquifer, and adequate amount of rainfall) in the coastal region are favourable for ASR to be an alternative fresh water source, particularly for dry season supply. The geological nature of the Asian deltas (Alekseev and Takaya, 1967; Goodbred and Kuehl, 2000; Ta et al., 2002; Tanabe et al., 2003a; Tanabe et al., 2006; Tanabe et al., 2003b) indicates that in most of the coastal areas shallow aquifer is semi-confined to fully confined by a silt-clay or clay layer. The shallower depth of the aquifer zone, found within 20 to 30 m below ground level, would reduce the development cost of ASR significantly. Using global elevation model data (EROS, 2002) we find that more than 120,000 km2 areas in Asian meg-deltas are very flat indicated by surface gradient $\leq$0.001 (Fig. S10). The low surface gradient could be a proxy for low groundwater head gradient in shallow aquifer, and given the geology is suitable in those areas, ASR could potentially be developed.

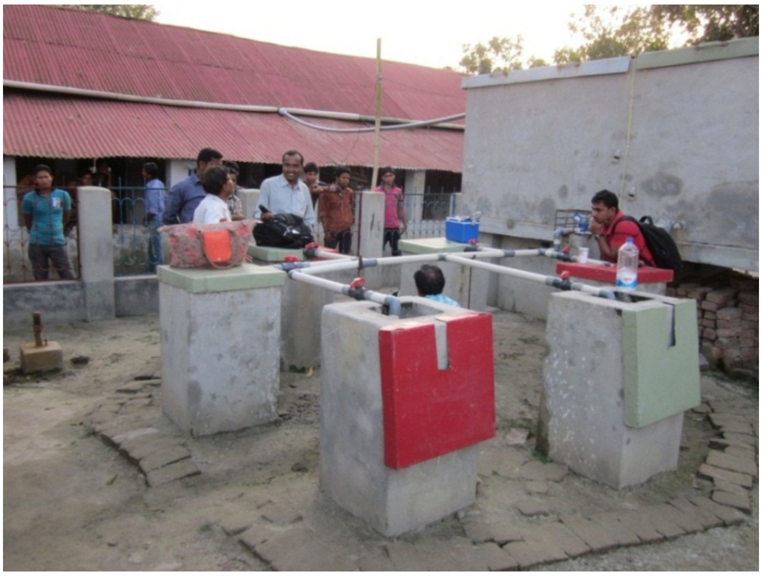

Figure S9: A managed aquifer recharge (ASR) site in southern Bangladesh. A recent intervention, in southwest Bangladesh, to the drinking water salinity problem is ASR. The aim is to store rainwater (with occasional inputs from ponds to meet the functional requirement of the system) in relatively shallow aquifers for dry-season use (Ahmed et al., 2010; Barker, 2013; Hasan, 2012). This approach also has the potential to protect the resource from contamination from storm surges, provided the injection and abstraction wells are capped prior to inundation or staying above the inundation. It therefore provides some resilience against these events. However, its long term sustainability and health impacts are yet to be known.

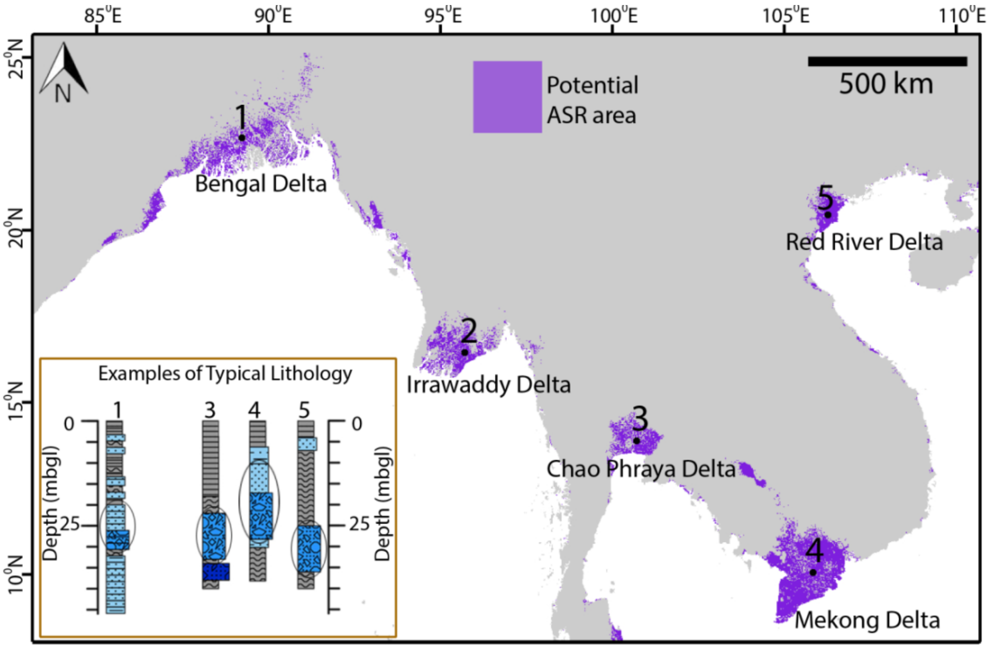

Figure S10: Extent of areas potential for ASR scheme in Southeast Asian deltas. Hydogeology is similar to the examples shown in the inset. Log 1 (Ahmed KM, unpublished data) is from an actual ASR site in southern Bangladesh. Water storing depth is indicated by an oval, and potential depths for such are indicated in others. Sources of logs: 3 (Alekseev and Takaya, 1967), 4 (Ta et al., 2002), 5 (Tanabe et al., 2006). Note log for Irrawaddy delta was not available but should be similar. In the logs, clays and silts, and sands are indicated by grey, and blue colours respectively.

References cited

Ahmed, K.M., Hasan, K., Sultana, S., Groen, K., Tuinhof, A., Spruijt, H., Patra, L., Ghosh, S.K., Huq, I., 2010. Action Research for Managed Aquifer Recharge in Bangladesh to Secure Groundwater Quantity and Quality ISMAR7: The 7th Annual International Symposium on Managed Aquifer Recharge (World Achieving Water Supply Sustainability and Reliability Through Managed Aquifer Recharge), Abu Dhabi, UAE.

Al-Salek, J.A., 1998. Coastal trapping and funneling effects on storm surges in the Meghna estuary in relation to cyclones hittin Noakhali-Cox's Bazar coast of Bangladesh. Journal of Physical Oceanography 28, 227–249.

Alam, M.M., Hossain, M.A., Shafee, S., 2003. Frequency of Bay of Bengal cyclonic storms and depressions crossing different coastal zones. International Journal of Climatology 23, 1119-1125.

Alekseev, M.N., Takaya, Y., 1967. An Outline of the Upper Cenozoic Deposits in the Chao Phraya Basin, Central Thailand. Southeast Asian Studies, Kyoto University 5, 334–352.

Anderson, H.R., 1978. Hydrogeologic Reconnaissance of the Mekong Delta in South Vietnam and Cambodia. United States Geological Survey, Washington DC., p. 24.

Barker, J.B., 2013. Numerical evaluation of community-scale aquifer storage, transfer and recovery technology, Department of Civil and Environmental Engineering. The University of Western Ontario, London, Ontario, Canada, p. 98.

Buschmann, J., Berg, M., Stengel, C., Winkel, L., Sampson, M.L., Trang, P.T.K., Viet, P.H., 2008. Contamination of drinking water resources in the Mekong delta floodplains: Arsenic and other trace metals pose serious health risks to population. Environment International 34, 756-764.

Chotamonsak, C., Salathé, E.P., Kreasuwan, J., Chantara, S., Siriwitayakorn, K., 2011. Projected climate change over Southeast Asia simulated using a WRF regional climate model. Atmospheric Science Letters 12, 213-219.

Delta Alliance, 2011. Vietnam-Netherlands Mekong Delta Masterplan project report (Mekong delta water resources assessment studies). Deltares, p. 68.

DPHE/DANIDA, 2001. Hydrogeology summary report Five Districts Water Supply and Sanitation Group (5DWSG), DPHE-DANIDA Water Supply and Sanitation Components, Bangladesh,. Department of Public Health Engineering (DPHE) and Danish International Development Assistance (DANIDA), Dhaka, p. 128.

Fritz, H.M., Blount, C.D., Thwin, S., Thu, M.K., Chan, N., 2009. Cyclone Nargis storm surge in Myanmar. Nature Geosci. 2, 448–449.

Goodbred, S.L., Jr., Kuehl, S.A., 2000. The significance of large sediment supply, active tectonism, and eustasy on margin sequence development: Late Quaternary stratigraphy and evolution of the Ganges-Brahmaputra delta. Sediment. Geol. 133, 227–248.

Hasan, M.M., 2012. Investigations on Groundwater Buffering in Khulna-Satkhira Coastal Belt Using Managed Aquifer Recharge, Department of Geology. University of Dhaka, Dhaka, Bangladesh, p. 85.

Hijioka, Y., Lin, E., Pereira, J.J., Corlett, R.T., Cui, X., Insarov, G.E., Lasco, R.D., Lindgren, E., Surjan, A., 2014. Asia, In: Barros, V.R., Field, C.B., Dokken, D.J., Mastrandrea, M.D., Mach, K.J., Bilir, T.E., Chatterjee, M., Ebi, K.L., Estrada, Y.O., Genova, R.C., Girma, B., Kissel, E.S., Levy, A.N., MacCracken, S., Mastrandrea, P.R., White, L.L. (Eds.), Climate Change 2014: Impacts, Adaptation, and Vulnerability. Part B: Regional Aspects. Contribution of Working Group II to the Fifth Assessment Report of the Intergovernmental Panel of Climate Change. Cambridge University Press, Cambridge, United Kingdom and New York, NY, USA, pp. XXX-YYY.

IPCC, 2007. Climate Change 2007: Impacts, Adaptation and Vulnerability. Contribution of Working Group II to the Fourth Assessment Report of the Intergovernmental Panel on Climate Change (IPCC). http://www.ipcc.ch.

Islam, A., Bala, S., Hussain, M., Hossain, M., Rahman, M., 2011. Performance of Coastal Structures during Cyclone Sidr. Natural Hazards Review 12, 111-116.

Johns, B., Ali, M.A., 1980. The numerical modelling of storm surges in the Bay of Bengal. Quarterly Journal of the Royal Meteorological Society 106, 1-18.

Karim, M.F., Mimura, N., 2008. Impacts of climate change and sea-level rise on cyclonic storm surge floods in Bangladesh. Global Environmental Change 18, 490-500.

Khan, A.E., Ireson, A., Kovats, S., Mojumder, S.K., Khusru, A., Rahman, A., Vineis, P., 2011. Drinking Water Salinity and Maternal Health in Coastal Bangladesh: Implications of Climate Change. Environ. Health Perspect. 119, 1328-1332.

Lee, T.-Y., Lawver, L.A., 1995. Cenozoic plate reconstruction of Southeast Asia. Tectonophysics 251, 85-138.

Løvholt, F., Glimsdal, S., Harbitz, C.B., Zamora, N., Nadim, F., Peduzzi, P., Dao, H., Smebye, H., 2012. Tsunami hazard and exposure on the global scale. Earth-Science Reviews 110, 58-73.

Mallick, B., Rahaman, K., Vogt, J., 2011. Coastal livelihood and physical infrastructure in Bangladesh after cyclone Aila. Mitig Adapt Strateg Glob Change 16, 629-648.
McIvor, A.L., Spencer, T., Möller, I., Spalding, M., 2012. Storm surge reduction by mangroves. The Nature Conservancy and Wetlands International, p. 35.
Murty, T.S., Flather, R.A., Henry, R.F., 1986. The storm surge problem in the bay of Bengal. Progress in Oceanography 16, 195-233.
NOAA-ESRL, 2011. GPCC Full Data Reanalysis Version 6.0 (at 0.5°, 1.0°, 2.5°): Monthly Land-Surface Precipitation from Rain-Gauges built on GTS-based and Historic Data. Available at http://www.esrl.noaa.gov/psd/ from Physical Sciences Division. National Oceanic & Atmospheric Administration (NOAA) and Earth System Research Laboratory (ESRL), Boulder Colorado.
NOAA, 2005. Saffir/Simpson scale. Tropical Prediction Center, National Hurricane Center, National Oceanic and Atmospheric Administration (NOAA), Washington, D.C.
Ortega, L., 2009. Saline intrusion study of South West Bangladesh, Civil and Environmental Engineering. Imperial College London, London, p. 80.
Qian, W., Lee, D.-K., 2000. Seasonal march of Asian summer monsoon. International Journal of Climatology 20, 1371-1386.
Ravenscroft, P., McArthur, J.M., Hoque, M.A., 2013. Stable groundwater quality in deep aquifers of Southern Bangladesh: The case against sustainable abstraction. Sci. Total Environ. 454–455, 627-638.
Resio, D.T., Westerink, J.J., 2008. Modeling the physics of storm surges. Physics Today 61, 33–38.
Saito, Y., Chaimanee, N., Jarupongsakul, T., Syvitski, J.P.M., 2007. Shrinking megadeltas in Asia: Sea-level rise and sediment reduction impacts from case study of the Chao Phraya delta, Inprint Newsletter of the IGBP/IHDP, Land Ocean Interaction in the Coastal Zone 2007/2, pp. 3-9.
Schneider, U., Becker, A., Finger, P., Meyer-Christoffer, A., Rudolf, B., Ziese, M., 2011. GPCC Full Data Reanalysis Version 6.0 at 0.5°: Monthly Land-Surface Precipitation from Rain-Gauges built on GTS-based and Historic Data, doi: 10.5676/DWD_GPCC/FD_M_V6_050.
Siddiki, U.R., Islam, M.N., Ansari, M.N.A., 2012. Cyclonic track analysis using GIS over the Bay of Bengal. Int. Journal of Applied Sciences and Engineering Research 1, 689–701.
Ta, T.K.O., Nguyen, V.L., Tateishi, M., Kobayashi, I., Tanabe, S., Saito, Y., 2002. Holocene delta evolution and sediment discharge of the Mekong River, southern Vietnam. Quaternary Science Reviews 21, 1807-1819.
Tanabe, S., Saito, Y., Lan Vu, Q., Hanebuth, T.J.J., Lan Ngo, Q., Kitamura, A., 2006. Holocene evolution of the Song Hong (Red River) delta system, northern Vietnam. Sediment. Geol. 187, 29-61.
The World Bank Group, 2010. Bangladesh: Economics of Adaptation to Climate Change. The World Bank Group, Washington, D.C. 20433 USA, p. 102.
Tripartite Core Group, 2008. Post-Nargis Periodic review I. United Nations (UN) in Myanmar, Myanmar, p. 107.
van Weert, F., van der Gun, J., Reckman, J., 2009. Global Overview of Saline Groundwater Occurrence and Genesis. International Groundwater Resources Assessment Centre (IGRAC), The Netherlands, p. 104.
Voris, H.K., 2000. Maps of Pleistocene sea levels in Southeast Asia: shorelines, river systems and time durations. Journal of Biogeography 27, 1153-1167.
Zheng, F., Westra, S., Sisson, S.A., 2013. Quantifying the dependence between extreme rainfall and storm surge in the coastal zone. Journal of Hydrology 505, 172-187.
